# Chiral Optical Tamm States at the Interface between a Dye-Doped Cholesteric Liquid Crystal and an Anisotropic Mirror

**DOI:** 10.3390/ma13153255

**Published:** 2020-07-22

**Authors:** Anastasia Yu. Avdeeva, Stepan Ya. Vetrov, Rashid G. Bikbaev, Maxim V. Pyatnov, Natalya V. Rudakova, Ivan V. Timofeev

**Affiliations:** 1 Kirensky Institute of Physics, Federal Research Center KSC SB RAS, 660036 Krasnoyarsk, Russia; S.Vetrov@inbox.ru (S.Y.V.); bikbaev@iph.krasn.ru (R.G.B.); MaksPyatnov@yandex.ru (M.V.P.); Natalya-V-Rudakova@iph.krasn.ru (N.V.R.); tiv@iph.krasn.ru (I.V.T.); 2 Siberian Federal University, 660041 Krasnoyarsk, Russia

**Keywords:** localization of light, photonic crystals, chirality, dye-doped cholesteric liquid crystal, optical Tamm states, resonant frequency dispersion

## Abstract

The resonant splitting of optical Tamm state numerically is demonstrated. The Tamm state is localized at the interface between a resonant chiral medium and a polarization-preserving anisotropic mirror. The chiral medium is considered as a cholesteric liquid crystal doped with resonant dye molecules. The article shows that the splitting occurs when dye resonance frequency coincides with the frequency of the Tamm state. In this case the reflectance, transmittance, and absorptance spectra show two distinct Tamm modes. For both modes, the field localization is at the interface between the media. The external field control of configurable optical and structural parameters paves the way for use in tunable chiral microlaser.

## 1. Introduction

Recently, there have been an increasing number of fundamental and applied works devoted to searching for new promising materials and designing the structures that exploit new ways of controlling light. Of particular interest is the optical Tamm state (OTS), i.e., a surface state localized at the interface between two media serving as mirrors, which does not transfer energy along the interface and exponentially decreases with increasing distance on either side of the interface [1,2,3]. The OTS is an electromagnetic analog of the Tamm state of electrons at the superlattice boundary [4]. In experiments, the OTS manifests itself as a narrow peak in the energy spectra of a sample [4,5]. The interest in the OTSs is due to the potential of their application in lasers and emitters [6,7,8], absorbers [9,10], sensors [11,12], as well as in photovoltaics [13], topological photonics [14,15], and other devices [16,17,18,19].

It appeared a nontrivial task to induce the OTS at the interface between an isotropic medium and a chiral medium, such as a cholesteric liquid crystal (CLC). A CLC is formed by oriented elongated molecules with the preferred direction twisted in space in the form of a helix. The CLCs are characterized by the continuous helical symmetry of the permittivity tensor and, due to its periodicity, represent one-dimensional photonic crystals [20]. The CLCs attract attention by their high sensitivity to electric and magnetic fields and temperature variation [21]. The qualitative difference between the CLCs and other types of photonic-crystal structures is that the former exhibit the diffraction selectivity to the polarization of light. Therefore, to localize a surface state at the interface between a CLC and an isotropic mirror, which does not preserve the polarization of light upon reflection, it is necessary to compensate the polarization variation using a quarter-wave plate [22], an additional anisotropic layer [23], or a chirality-preserving mirror [24]. Such mirror can preserve not only the chirality sign, but also the value of the ellipticity of the incident radiation. This particular case can be called a polarization-preserving anisotropic mirror (PPAM) or a mirror that does not change the sign of polarization of the reflected light [25]. The simplest example of such structures is a pile of identical anisotropic layers with alternating orientations of the optical axes. This structure was studied by Reusch [26] as a polarization filter. Due to the chiral properties of the Reusch pile, the incident wave of one circular polarization passes through the structure, while the component of the other polarization is reflected. This property is observed both at the normal [27] and oblique incidence of light [28]. A special class is the equichiral Reusch piles, in which the optical axes of neighboring layers become perpendicular to each other [29]. Previously, we demonstrated the possibility of implementing the OTS at the interface between a PPAM and a CLC; the obtained localized surface state was called the chiral optical Tamm state (COTS) [23,24,30,31].

Liquid crystals doped with various micro- and nanoparticles [32] or dye molecules evoke great interest, since they combine the fluidity, crystal anisotropy, and specific properties of dye particles or molecules. In a dye-doped CLC, a distributed feedback lasing with the lowest laser pumping threshold can be implemented [33,34,35,36]. The presence of dye molecules can lead to the qualitative rearrangement of the band structure of the CLC spectrum, specifically, to splitting of the photonic band gap (PBG) into several PBGs [37]. Embedding of a resonant defect layer doped with metal nanoparticles into a CLC adds new features to the spectral and polarization properties of the latter [38].

In view of the persistent popularity of the discussed topic, we set a problem to examine the spectral properties of the COTS localized at the interface between a PPAM and a dye-doped cholesteric liquid crystal (DDCLC). The advantage of the proposed structure is the significant expansion of the possibility of effective controlling the parameters of the photon energy spectrum and the transmittance, reflectance, and absorptance spectra of the structure. A fundamentally new effect of COTS splitting at the interface between the media at the coinciding dye and COTS resonant frequencies was established. In this case, two energy levels corresponding to two new COTSs were observed at the intersection of the DDCLC and PPAM band gaps. The complete or partial overlap of the band gaps of CLC and PPAM in the energy spectrum is a condition for the COTS formation at the two environments border plane. In this case, the energy level occurs at the overlap of the band gaps corresponding to the chiral optical Tamm mode localized at the interface of the environments. The dye molecules included in the CLC are influenced by an electromagnetic field located near the environments border. The resonant mode of the mutual influence of COTS and the resonance of the dye molecules lead to the split of the COTS frequency. The splitting effect depends significantly on the concentration of dye molecules as well as on the step of the CLC helix. When reconstructing from the resonance, the splitting effect of COTS is persistent as long as the COTS lies in the frequency dispersion of the dye molecules.

## 2. Description of the Model

**Figure 1:**
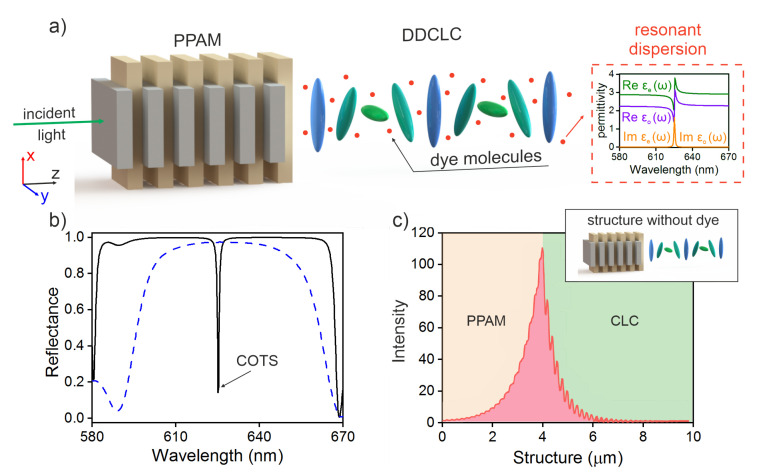
(**a**) Schematic of the structure consisting of a PPAM with length *d* and a DDCLC with length *L*. Inset: imaginary (yellow curve) and real (green and purple curves) are parts of the effective permittivity component of the DDCLC tensor. (**b**) Reflectance spectra of the PPAM-CLC structure. The solid curve corresponds to the diffracting polarization, i.e., the circular polarization that is reflected from CLC and the dashed curve, to the nondiffracting one. The PPAM length is d=3.92 μm, the period is $\Lambda =d_{V}+d_{H}$, $d_{V}=100$ nm, $d_{H}=96$ nm, the number of periods is $N_{PPAM}=20$, and the refractive indices are $n_{e}^{p}=1.7$ and $n_{o}^{p}=1.5$. The CLC layer length is L=5.85 μm, the number of periods is $N_{CLC}=30,$ the helix pitch is p=390 nm, and the extraordinary and ordinary refractive indices are $n_{e}=1.7$ and $n_{o}=1.5$. The CLC band gap center is $\lambda_{0}=625$ nm and ϕ=π/4. (**c**) Spatial distribution of the local field intensity in the sample corresponding to a COTS wavelength of λ=625.3 nm normalized to the initial value.

The investigated finite structure schematically shown in Figure 1a consists of a PPAM conjugated with a right-handed DDCLC. The multilayer PPAM structure consists, in its turn, of alternating uniaxial dielectric layers with different refractive indices $n_{e}^{p}=\sqrt{\varepsilon_{e}^{p}}$ ${\mathrm{and}\;n}_{o}^{p}=\sqrt{\varepsilon_{o}^{p}}$ and it is characterized by dielectric tensors of two neighboring layers, which can be written as:

(1) $${\hat{\varepsilon }}_{V}=\left(\begin{array}{ccc} 1.7 & 0 & 0\\ 0 & 1.5 & 0\\ 0 & 0 & 1.7 \end{array}\right),\;{\hat{\varepsilon }}_{H}=\left(\begin{array}{ccc} 1.5 & 0 & 0\\ 0 & 1.7 & 0\\ 0 & 0 & 1.7 \end{array}\right).$$

In should be noted what the optical axes of the PPAM are directed along *x* (yellow layer) and *y* (grey layer) axis respectively (see Figure 1a). The number of unit cells (the number of V-H pairs) in the structure is $N_{PPAM}$, the period of the structure is $\Lambda =d_{V}+d_{H}$, where $d_{V}$ and $d_{H}$ are the thicknesses of the unit cell layers; and the PPAM thickness is $d=N_{PPAM}\times \Lambda$. The CLCs with a continuous helical symmetry of the permittivity tensor are characterized by the helix pitch *p*, the cholesteric layer thickness *L*, the number of periods $N_{CLC},$ and the ordinary and extraordinary refractive indices $n_{\|}=n_{e}=\sqrt{\varepsilon_{\|}}$ and $n_{⊥}=n_{o}=\sqrt{\varepsilon_{⊥}}$. The structure under study is limited to a medium with the refractive index $(n_{e}+n_{o})/2.$

We consider the normal incidence of light onto the structure. The angle between the CLC director and the optical axis of the PPAM layer conjugated with a CLC is denoted by φ. If light propagates along the axis, the CLC permittivity and permeability tensors are:

(2) $$\hat{\varepsilon }\left(z\right)=\varepsilon_{m}\left(\begin{array}{ccc} 1+\delta \cos(qz) & \pm \delta \sin(2qz) & 0\\ \pm \delta \sin(qz) & 1-\delta \cos(qz) & 0\\ 0 & 0 & 1-\delta \end{array}\right),\;\hat{\mu }\left(z\right)=\hat{I},$$

respectively, where q=4π/p, $\varepsilon_{m}=\left(\varepsilon_{e}+\varepsilon_{o}\right)/2$ and $\delta =\left(\varepsilon_{e}-\varepsilon_{o}\right)/\left(\varepsilon_{e}+\varepsilon_{o}\right).$

It is supposed that the order parameter that characterizes the degree of the dipole moment order of the dye molecules transition is zero. This corresponds to the chaotic orientation of the dipole moment of the dye molecules transition. In this case, the presence of dye molecules in the CLC matrix causes the frequency dependence of the main values of the local dielectric tensor, and we assume the Lorentz form of this frequency dependence [37]. In addition, a small volume concentration of the dye molecules in the CLC matrix is suggested, since the coupling of oscillators through a local field is not taken into account. The characteristic concentrations of the dye molecules correspond to the concentration of molecules in an ideal gas under normal conditions. Lorentz form of their frequency dependence:

(3) $$\varepsilon_{e}\left(\omega \right)=\varepsilon_{e}+\frac{f_{1}}{\omega_{01}^{2}-\omega^{2}-i\gamma_{1}\omega },$$

(4) $$\varepsilon_{o}\left(\omega \right)=\varepsilon_{o}+\frac{f_{2}}{\omega_{02}^{2}-\omega^{2}-i\gamma_{2}\omega },$$

where $\gamma_{1}$ and $\gamma_{2}$ are the damping coefficients, $\omega_{01}$ and $\omega_{02}$ are the resonant frequencies, $f_{1}$ and $f_{2}$ are the quantities proportional to the oscillator strength ${\bar{f}}_{1,2}$.

(5) $$f_{1,2}=\frac{4\pi Ne^{2}}{m}\times {\bar{f}}_{1,2}.$$

In Equation (5), *N* is the number of dye molecules in the unit volume; the dipole oscillator strength ${\bar{f}}_{1,2}$ is, as a rule, about tenths of unity; and *e* and *m* are the electron charge and mass, respectively. Hereinafter in the calculation, we assume $f_{1}=f_{2}=f$, $\gamma_{1}=\gamma_{2}=\gamma$, $\omega_{01}=\omega_{02}=\omega_{0}$.

The possible way of fabrication of the proposed structure is to use polymer-stabilized liquid crystals [35,39,40,41]. Dye-doped polymeric cholesteric liquid crystal films can be made in the same manner as Shmidtke et al. [35] and Jeong et al. [41] who studied defect mode lasing in such films. PPAM can be created from polymerized nematic layers, for example 5CB. Each layer of LC should be applied sequentially and polymerized. Despite the use of polymerized materials, the possibility of manipulating CLCs remains [42,43].

A numerical analysis of the spectral properties of the system and the field distribution in the sample of a dye-doped cholesteric conjugated with an anisotropic mirror is performed using the Berreman 4 × 4 transfer matrix method [44]. The equation describing the propagation of light at frequency ω along the *z* axis normal to the structural layers has the form:

(6) $$\frac{d\psi }{dz}=\frac{i\omega }{c}\Delta \left(z\right)\psi \left(z\right),$$

where $\psi \left(z\right)={\left(E_{x},H_{y},E_{y},-H_{x}\right)}^{T}$ and Δ(z) is the Berreman matrix, which depends on the dielectric function and the incident wave vector.

## 3. Results and Discussion

Figure 1b,c, show the reflectance spectra and the local field intensity distribution at wavelength of COTS in the PPAM-CLC sample without dye molecules. It should be noted that, at the equal PPAM and CLC refractive indices, the CLC band gap lies in the wavelength range of 580–670 nm, which is 20 nm higher than the PPAM band gap with the boundaries from 595 to 665 nm. It is well-known that the complete or partial overlap of the band gaps in the energy spectrum is a condition for the formation of a COTS at the interface between two media. The fulfillment of this condition ensures the excitation of a localized state at the diffracting polarization at a wavelength of λ=625.3 nm, while at the polarization of the opposite sign (nondiffracting polarization), the COTS is not excited. The observed state is high-*Q* and can be effectively tuned in frequency [30].

**Figure 2:**
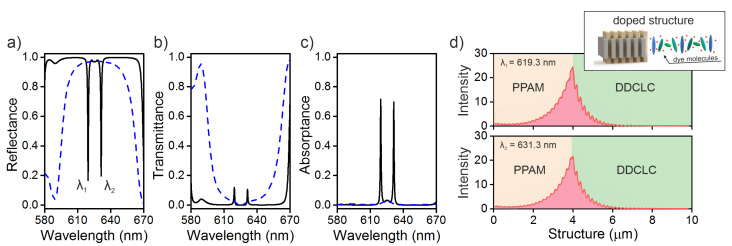
(**a**) Reflectance, (**b**) transmittance, and (**c**) absorptance spectra of the PPAM-DDCLC structure. The solid curve corresponds to the diffracting polarization and the dashed curve, to the nondiffracting one. The damping parameters are $\gamma =4\times 10^{12}$ s${}^{-1}$ and $f=2\times 10^{28}$ s${}^{-2}$. The rest parameters are the same as in Figure 1b. (**d**) Spatial distribution of the local field intensity in the sample normalized to the initial value.

We add the CLC with fluorescent dye molecules, which have parameters of $\gamma =4\times 10^{12}$ s${}^{-1}$ and $f=2\times 10^{28}$ s${}^{-2}$. In this case, the principal values of the CLC local dielectric tensor become frequency-dependent. If the resonant frequency of dye molecules coincides with the COTS frequency, then two modes, instead of one, appear in the spectra. These modes yield dips at wavelengths of $\lambda_{1}=619.3$ nm and $\lambda_{2}=631.3$ nm (see Figure 2). The splitting value is Δλ=12 nm. At an oscillator strength of $\bar{f}\approx 0.5$, Equation (5) yields an estimated number of $N\approx 10^{19}$ cm${}^{-3}$ of dye molecules in the unit volume.

Figure 2d shows the spatial distributions of the COTS local field intensity at wavelengths of 619.3 and 631.3 nm, which correspond to the reflection maxima in the spectrum (Figure 2a). It can be seen that the light is localized at the PPAM-DDCLC interface and the local field intensity decreases exponentially with increasing distance from the interface. There are two different kinds of the local intensity rippling inside PPAM and DDCLC.

First, the PPAM rippling is due to nonhomogeneity of PPAM material. The layers are virtually quarter-vawelength. The multiple layer boundaries correspond to standing wave nodes and antinodes of x and y components of electric field [30], that results in rippling of the overall field. Second, the local intensity rippling in CLC has different origin, because the CLC material is homogeneously twisted along z direction and its main eigenwave is a smoothly evanescenting exponential [20]. In total there are four eigenwaves inside the CLC, and all of them are excited with the amplitudes depending on boundary conditions and excitation wave polarization. The interference of eigenwaves results in some rippling, small minima and maxima of the overall field.

The *Q* factor of the two modes obtained by splitting, at the resonant frequency of dye molecules coinciding with the COTS center, will be approximately the same and equal to 774 for the high-frequency mode and 702 for the low-frequency mode. At the detuning of the resonant frequency from the COTS wavelength, one can enhance the *Q* factor of one peak by reducing the *Q* factor of the other. Below, we show that the *Q* factor of the new COTSs obtained by the splitting can be significantly increased by changing the parameters of dye molecules.

**Figure 3:**
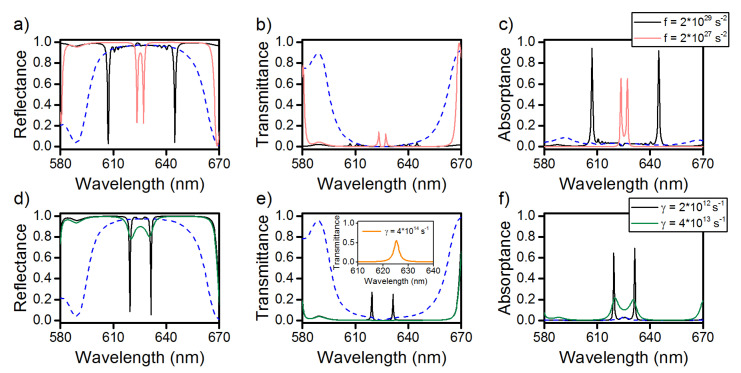
(**a**,**d**) Reflectance, (**b**,**e**) transmittance, and (**c**,**f**) absorptance spectra of the investigated structure at different volume concentrations of dye molecules and damping coefficients. Solid curves correspond to the diffracting polarization and the dashed curve, to the nondiffracting one. The rest parameters are the same as in Figure 2. The insert in figure (**e**) shows the transmission spectra of the structure for $\gamma =4\times 10^{14}$ s${}^{-1}$.

Let us consider the effect of the dye molecule concentration on the splitting value and the COTS position. Figure 3a–c show that, with an increase in the dye molecule concentration by an order of magnitude, two COTSs arise at wavelengths of 607 and 644.9 nm, respectively, and the splitting value increases by 25.9 nm and attains Δλ=37.9 nm. As the concentration of fluorescent molecules decreases by an order of magnitude, the COTSs are observed at wavelengths of 623.4 and 627.1 nm and the splitting value decreases down to Δλ=3.7 nm.

Figure 3d–f illustrate the sensitivity of the COTS splitting to the changes in the damping coefficient. We do not specify the physical origins of the damping, which can be very different. As the γ value decreases by two times, the COTSs appear in the spectra at wavelengths of 619.3 and 631.3 nm and the *Q* factor of the split modes grows to about 1000. In this case, the splitting value remains the same and amounts to Δλ=12 nm. As the γ value increases by an order of magnitude, the new COTSs become poorly distinguishable, the peak maxima corresponding to wavelengths of 620.2 and 630.3 nm approach each other, and the splitting value decreases to Δλ=10.1 nm. We would like to note that, at $\gamma =4\times 10^{14}$ s${}^{-1}$, the splitting effect does not manifest itself, and the low-*Q* dip at a wavelength of 625.3 nm is observed in the reflectance spectrum, which corresponds to the COTS wavelength in the PPAM-CLC structure without dye.

**Figure 4:**
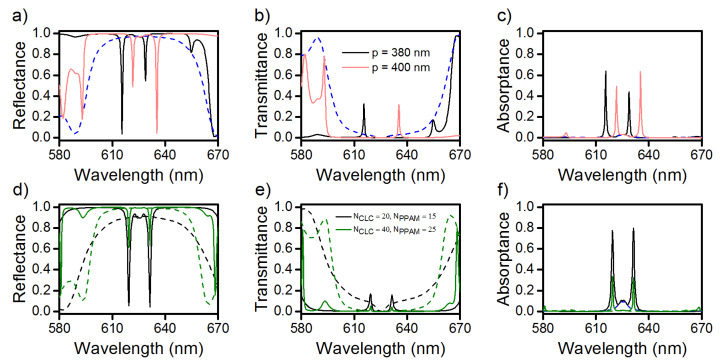
(**a**,**d**) Reflectance, (**b**,**e**) transmittance, and (**c**,**f**) absorptance spectra of the investigated structure at different cholesteric helix pitches and PPAM and CLC geometric parameters. Solid lines correspond to the diffracting polarization and the dashed lines, to the nondiffracting one. The rest parameters are the same as in Figure 2.

As was mentioned above, an important advantage of the CLCs over other types of photonic crystals is their high sensitivity to external fields. A strong dependence of the helix pitch, for example, on temperature or applied voltage can be used to effectively control the COTS splitting value. Thus, a decrease in the helix pitch by 10 nm leads to an increase in the splitting value by 1.2 nm to Δλ=13.2 nm (Figure 4a–c). In this case, as can be seen in Figure 4a,c, the positions of the COTS frequencies shift to the short-wavelength spectral region by 615.6 and 628.8 nm, respectively. In the transmittance spectrum shown in Figure 4b, due to the damping, the only short-wavelength localized peak at a wavelength of 615.6 nm remains. With an increase in the helix pitch by 10 nm, the opposite situation is implemented. It can be seen from the reflectance and absorptance spectra shown in Figure 4a,c that, with an increase in the helix pitch to 400 nm, the positions of the COTS frequencies shift to the long-wavelength spectral region to 621.7 and 635.5 nm and the splitting value increases to Δλ=13.5 nm. In the transmittance spectrum in Figure 4b, the only long-wavelength COTS remains localized at a wavelength of 635.5 nm.

Figure 4d–f illustrate the possibility of controlling the spectral properties of the COTS by optimizing the geometric parameters of the PPAM-DDCLC structure. Let us reduce the size of the structure by decreasing the $N_{PPAM}$ value from 20 to 15 and the $N_{CLC}$ value from 30 to 20. In this case, the spectral positions of the COTS frequencies do not change, i.e., the splitting value remains unchanged, while the *Q* factor of the peaks decreases. At the increasing sample length, at $N_{CLC}=40$ and $N_{PPAM}=25$, due to the increased band gap contrast, the gaps in the reflectance spectrum are about 38% and the corresponding COTS peaks in the transmittance spectrum are almost absent, due to an increase in the length of examined sample.

**Figure 5:**
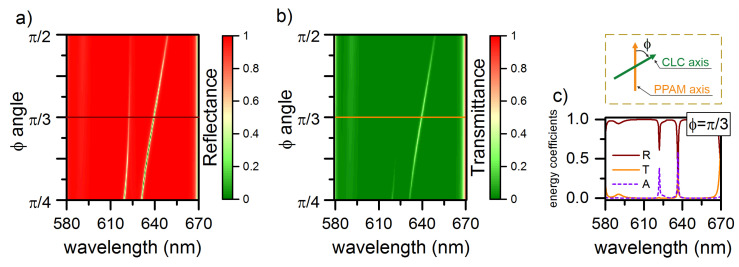
(**a**) Reflectance and (**b**) transmittance spectra of the structure at different angles φ. (**c**) Reflectance, transmittance, and absorptance spectra of the structure at φ=π/3. Insert: Definition of angle ϕ as the angle between the CLC director at the boundary and the optical axis of the PPAM layer conjugated with the CLC. The rest parameters are the same as in Figure 2.

The similar effects can be implemented in a different way, i.e., by changing the angle φ between the PPAM and DDCLC optical axes at the interface between the media (inset in Figure 5). When passing the interface between two mirrors, the geometric phase is controlled by rotation of the mirrors in the interface plane. Figure 5a,b show that, in the reflectance and transmittance spectra, at the diffracting polarization, depending on the angle φ, the reflection of the right-hand peak decreases from 24% to 8%, while the transmittance increases from 11% to 50%, with an increase in ϕ from π/4 to π/3. Upon further rotation of the angle, the transmittance of the long-wave peak decreases and the reflectance increases. We would like to note that, when ϕ is rotated in the opposite direction, the inverse situation is observed in the transmittance spectrum, when the transmittance is only preserved for the short-wave COTS.

Figure 5c shows the reflectance, transmittance, and absorptance spectra of the structure at φ=π/3. In this case, the COTSs appear in the spectra at wavelengths of 622 and 636.4 nm. The splitting value increases by 4.4 nm to Δλ=16.4 nm and the transmittance spectrum only reflects the long-wavelength COTS. Note that when changing the helix pitch or angle φ, the position of the COTS slightly deviates from the value of 625.3 nm. In this case, the splitting effect will be preserved only if the frequency of COTS lies in the frequency dispersion range of the dye molecules.

## 4. Conclusions

Thus, the study of the properties of the PPAM-CLC-based model structures shows that doping of a CLC with dye molecules leads to the splitting of the COTS localized at the PPAM-DDCLC interface, if the resonant frequency of dye molecules coincides with the COTS frequency. As a result, the resonances corresponding to the two modes localized at the interface appear in the spectra. The possibility of effective controlling the spectral position and *Q* factor of these resonances, as well as the COTS splitting value, by changing the dye parameters and the CLC and PPAM geometric parameters was demonstrated. The proposed structure can be used to create miniature lasers with the circularly polarized fundamental mode, as well as to design narrow-band and tunable filters.

## Abbreviations

The following abbreviations are used in this manuscript:

OTS	Optical Tamm State
COTS	Chiral Optical Tamm State
PPAM	Polarization-Preserving Anisotropic Mirror
CLC	Cholesteric Liquid Crystal
DDCLC	Dye-doped Cholesteric Liquid Crystal
